# Systematic Review of Patient Preference Studies in Non-metastatic Breast Cancer Adjuvant Medication Therapy: Attribute Selection

**DOI:** 10.5812/ijpr-144877

**Published:** 2024-06-01

**Authors:** Ali Homayouni, Shekoufeh Nikfar, Fariborz Mokarian Rajabi, Mona Nili, Kimberly M. Kelly, Akbar Abdollahiasl

**Affiliations:** 1 Department of Pharmacoeconomics and Pharmaceutical Administration, School of Pharmacy, Tehran University of Medical Sciences, Tehran, Iran; 2 Department of Internal Medicine, School of Medicine, Isfahan University of Medical Sciences, Isfahan, Iran; 3 Pharmaceutical Systems and Policy, West Virginia University School of Pharmacy, Health Sciences Center, West Virginia, USA

**Keywords:** Breast Cancer, Patient Preferences, Discrete Choice Experiment, Attributes

## Abstract

**Context:**

Breast cancer poses significant challenges due to its high incidence and prevalence, necessitating heightened attention. Understanding how patients prioritize different treatment options based on various attributes can assist healthcare decision-makers in maximizing patient utility. The discrete choice experiment, a conjoint method, facilitates preference elicitation by presenting different attributes and choices. This systematic review aims to identify key factors in patient preference research related to adjuvant treatment for early breast cancer characterized by hormone receptor-positive, HER2-negative status.

**Evidence Acquisition:**

PubMed, Embase, Web of Science, and Scopus were searched from 01.01.2000 to 31.03.2023. Original English articles reporting patient preferences in adjuvant breast cancer treatment were retrieved based on predefined inclusion and exclusion criteria. Included studies were examined through a narrative synthesis approach, with descriptive statistics employed for analysis.

**Results:**

Out of 1163 articles reviewed, four met the inclusion criteria and were conducted in the USA, Canada, and the Netherlands. Attributes extracted from all studies included alopecia, sensory neuropathy, motor neuropathy, myalgia/arthralgia, nausea, vomiting, fatigue, neutropenia, mucositis/stomatitis, hand-foot syndrome, diarrhea, prevention of breast cancer recurrence, osteoporosis, risk of endometrial cancer, joint and muscle pain, fluid retention, libido decrease, hot flashes, ECG monitoring, efficacy, treatment regimen, 5-year invasive disease-free survival (iDFS), dosing schedule, and treatment duration. The most frequently reported attributes were side effects, efficacy, and treatment regimen. Systematic review was commonly used to determine which attributes and levels to include. The minimum number of attributes identified per study was seven, and the maximum was 12. Sample sizes ranged from 102 to 300, with none of the studies mentioning the method of sample size estimation. Ordinary Least Squares, logistic regression, and hierarchical Bayes regression were the most frequent analysis methods.

**Conclusions:**

Side effects, 5-year iDFS, and treatment regimen are three attributes identified for conducting discrete choice experiment studies. Utilizing conjoint analysis to assess patient preferences for breast cancer treatment can aid in selecting optimal treatment regimens and improving patient adherence. Moreover, adhering to guidelines for developing experimental designs and conducting data analysis is essential for yielding robust results when employing preference elicitation methods.

## 1. Context

Cancer ranks among the top three leading causes of death globally, following stroke and cardiovascular diseases (1). The Global Cancer Observatory (GCO) reported in 2020 that breast cancer is projected to be the most prevalent cancer and the leading cause of cancer-related mortality among women, with 2 261 416 new cases and 684 996 deaths (2).

With the emergence of new cancer treatments and the growing emphasis on patient preferences and shared decision-making, various preference assessment methods have been developed (3). These methods serve stakeholders such as patients, physicians, and policymakers (4, 5). Given that patients are central to the treatment process and their adherence to treatment protocols is crucial, understanding their preferences is essential for ensuring proper adherence.

Breast cancer manifests in different stages according to the tumor size, node, metastasis (TNM) staging algorithm, along with tumor markers such as human epidermal growth factor 2 (HER2) and HR (hormone receptor), which can influence the choice of treatment regimen (6). A considerable number of diagnosed breast cancer patients fall into the category of HER2-negative and HR-positive, making them candidates for chemotherapy or hormone therapy. Guidelines recommend selecting a medical treatment regimen based on factors like tumor size and the results of the Oncotype DX test for early-stage breast cancer patients with HER2-negative, HR-positive tumor markers (7). In situations where the Oncotype DX test is unavailable to clinicians, decision-makers such as physicians and patients are confronted with a selection scenario that necessitates weighing the risks and benefits of different treatment regimens. Understanding patients' preferences can assist clinicians (decision-makers) in making more informed decisions. Additionally, these findings can aid pharmaceutical companies in pinpointing the appropriate stage of product development to enhance patient adherence, and policymakers can leverage them to maximize patient utility (8).

The discrete choice experiment (DCE) is a stated-preference method that employs hypothetical scenarios to elicit respondents' preferences by prompting them to choose between them (9). Each scenario comprises various levels of pre-specified attributes, allowing respondents to trade-off between them based on their utility, which aims to be maximized (10). By examining previous patient preference research in the adjuvant treatment of hormone-positive, HER2-negative breast cancer, we can identify the crucial attributes and their corresponding levels.

## 2. Evidence Acquisition

This study's methodology was prespecified and documented based on the PRISMA protocol (11) in the PROSPERO database, registration number CRD42021240344.

### 2.1. Eligibility Criteria

Original articles eliciting patients' preferences using various methods (e.g., conjoint analysis, DCE, best-worst scaling) will be included. The search time interval is from January 1, 2000, to March 31, 2023. The target population includes individuals over 18 years old with non-metastatic breast cancer. Exclusion criteria include: (1) qualitative preference studies, (2) review articles, commentaries, abstracts, editorials, expert opinions, letters, and conference proceedings, (3) studies focusing solely on healthcare professionals' treatment preferences, (4) studies focusing solely on metastatic breast cancer therapy, (5) any language other than English.

### 2.2. Information Sources and Search

We conducted searches across electronic databases, including PubMed, Scopus, EMBASE, and Web of Science from January 1, 2000, to March 31, 2023. The search strategy used for PubMed is detailed in ESM 1.

### 2.3. Study Selection

**Figure 1.**
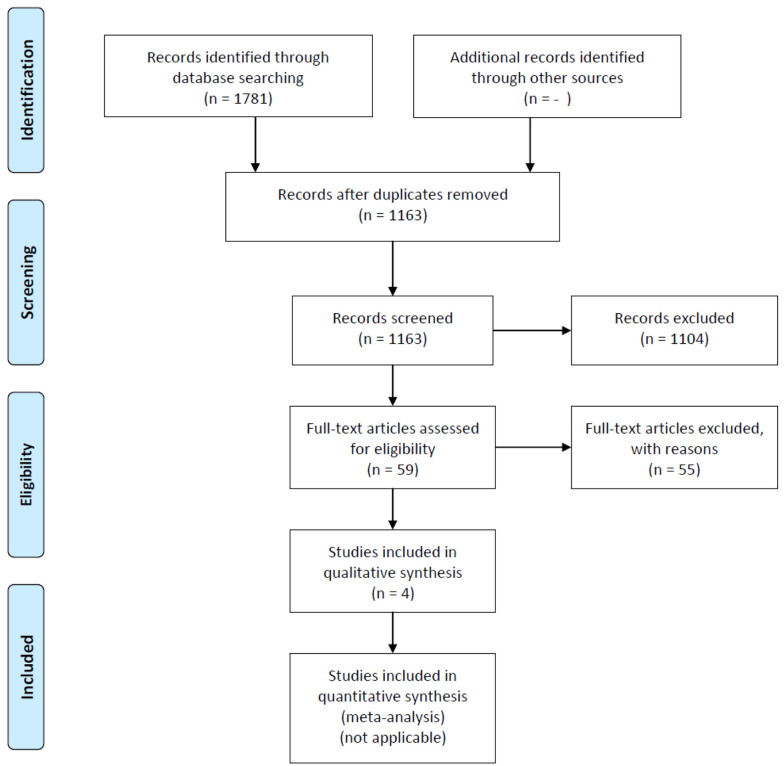
PRISMA flowchart for systematic review

Eligible articles were imported into Endnote software. After excluding duplicates, two independent reviewers assessed the title and abstract. Subsequently, two independent reviewers evaluated the full text of eligible articles, resolving any conflicts through consensus discussions with a third expert reviewer (Figure 1). 

### 2.4. Data Extraction and Synthesis of Results

Two independent reviewers extracted data from eligible articles based on the predetermined list in the systematic review protocol and entered it into an Excel sheet. The result table was completed after reviewing conflicts and reaching a consensus (Table 1). 

**Table 1.** Study Characteristics

No.	Study	Year	Country	Intervention	Title
**1**	Beusterien et al. (12)	2012	USA	Adults > 18 years; Stages 1 - 4; history of chemotherapy in the last five years	Patient preferences for chemotherapies used in breast cancer
**2**	Wouters et al. (13)	2013	Netherlands	Patients how are receiving hormone therapy	Trade-off preferences regarding adjuvant endocrine therapy among women with estrogen receptor-positive breast cancer
**3**	Beusterien et al. (14)	2014	Canada	Female breast cancer patients, with disease of any stage, who were currently receiving neo/adjuvant or palliative chemotherapy.	Use of Conjoint Analysis to Assess Breast Cancer Patient Preferences for Chemotherapy Side Effects
**4**	Beusterien et al. (15)	2021	USA	Adults > 18 years; Stage 2 or 3; history of chemotherapy in the last 5 years	Patient, Oncologist, and Payer Preferences for Adjuvant Endocrine Therapy and CDK4/6 Inhibitor Regimens in Early-Stage Breast Cancer: A Discrete Choice Experiment

### 2.5. Risk of Bias Assessment

The risk of bias assessment was performed based on a checklist from the Conjoint Analysis Applications in Health—a Checklist: “A Report of the ISPOR Good Research Practices for Conjoint Analysis Task Force” (4). The checklist included key components of the study such as the research question, attributes and levels, task construction, experimental design, preference elicitation, instrument design, data collection, statistical analyses, results and conclusions, and study presentation. Two research team members independently conducted this process, resolving any conflicts through consensus with a third party.

## 3. Results

### 3.1. Study Selection

After excluding duplicates from the 1781 extracted articles in the first step, the title and abstracts of 1163 articles were reviewed. Of these, 1104 articles were excluded based on the exclusion criteria. Subsequently, two independent reviewers examined the full text of 59 articles, of which 55 were excluded. Finally, four articles remained for data extraction (Figure 1). 

### 3.2. Study Characteristics

Table 2 provides an overview of the characteristics of the eligible studies. The studies were conducted in the Netherlands, Canada, and the USA. All studies used online interviews to gather data, with only one study employing both face-to-face and online interviews. Sample sizes ranged from 102 to 300 participants in each study. One study was limited to stage 2-3 cancer, while two included all stages. One study did not specify the stage of the target population. Preferences were evaluated in breast cancer patients based on the following characteristics: Patient preferences for chemotherapies used in breast cancer (16), preferences related to trade-offs in adjuvant endocrine therapy for women with estrogen receptor-positive breast cancer (13), the utilization of Conjoint Analysis to evaluate patient preferences for chemotherapy side effects (14), and a DCE exploring preferences among patients, oncologists, and payers for adjuvant endocrine therapy and CDK4/6 inhibitor regimens in early-stage breast cancer (15).

**Table 2.** Attributes and Levels

No.	Study	N Alternatives	N Attributes	Attributes and Levels	Attribute Selection
**1**	Beusterien et al. (12)	2	12 (3 - 6 levels)	(1) Alopecia: 0%, 48%, 94%; (2) Grade III/IV sensory neuropathy: 0%, 7%, 13%; (3) Grade III/IV motor neuropathy: 0%, 4%, 10%; (4) Grade III/IV myalgia/arthralgia: 0%, 4%, 15%; (5) Grade III/IV nausea and vomiting: 0%, 4%, 15%; (6) Grade III/IV fatigue: 0%, 8%, 24%; (7) Grade IV neutropenia resulting in hospitalization: 0%, 9%, 23%; (8) Grade III/IV mucositis/stomatitis: 0%, 5%, 10%; (9) Grade III/IV hand-foot syndrome: 0%, 5%, 12%; (10) Grade III/IV diarrhea: 0%, 5%, 15%; (11) Efficacy: Has not shown an additional survival benefit, Has shown an additional survival benefit of 1 month, Has shown an additional survival benefit of 3 months; (12) Regimen: Frequency and duration of chemotherapy administration: - 21-day cycle; oral tablets taken twice daily for the first 2 weeks: - 21-day cycle; 2 - 5-minute infusion on days 1 and 8; - 21-day cycle; 3-hour infusion on day 1; - 28-day cycle; 6 - 10-minute infusion on days 1, 8, and 15; - 21-day cycle; 30-minute infusion on days 1, 8, and 15; - 21-day cycle; 3-hour infusion on days 1, 8, and 15	Comprehensive literature review; The Common Toxicity Criteria grading system; Detailed assessment of breast cancer forum discussions; Consultation with clinical experts
**2**	Wouters et al. (13)	2	8 (2 levels)	(1) Prevention of breast cancer recurrence: In 3 of 10 women, in 5 of 10 women; (2) Osteoporosis: Lessens osteoporosis, Aggravates osteoporosis; (3) Risk of endometrial cancer: In 1 of 1000 women, In 5 of 1000 women; (4) Joint and muscle pain: A bit, Moderate to severe; (5) Fluid retention: A bit, Moderate to severe; (6) Libido decrease: A bit, Moderate to severe; (7) Hot flashes: Some per month, Some per week; (8) Regimen duration (Years of endocrine therapy use): 2 years, five years	literature review and online focus groups conducted with women treated with endocrine therapy
**3**	Beusterien et al. (14)	2	10 (3 - 7 levels)	(1) Alopecia: 12%, 46%, 90%; (2) Grade I/II peripheral neuropathy: 6%, 35%, 71%; (3) Grade III/IV Peripheral neuropathy: 1%, 10%, 21%; (4) Grade I/II motor neuropathy: 1%, 5%, 15%; (5) Grade III/IV motor neuropathy: < 1%, 2%, 5%; (6) Grade I/II myalgia: 8%, 23%, 44%; (7) Grade III/IV myalgia: 0%, 3%, 8%; (8) Grade I/II nausea: 27%, 42%, 50%; (9) Grade III/IV nausea: 2%, 4%, 9%; (10) Grade I/II fatigue: 18%, 34%, 55%; (11) Grade III/IV fatigue: 4%, 8%, 16%; (12) Neutropenia: 3%, 9%, 16%; (13) Grade I/II hand-foot syndrome: 0%, 47%, 64%; (14) Grade III/IV hand-foot syndrome: < 1%, 10%, 57%; (15) Grade I/II diarrhea: 0%, 13%, 30%; (16) Grade III/IV diarrhea: 0%, 5%, 15%; (17) Regimen: - 21-day cycle; oral tablets taken twice daily for the first 2 weeks: - 21-day cycle; 1-hour infusion on day 1; - 21-day cycle; 2- to 5-minute infusion on days 1 and 8; - 21-day cycle; 30-minute infusion on days 1 and 8; - 21-day cycle; 10-minute infusion on days 1, 8, and 15; - 21-day cycle; 30-minute infusion on days 1, 8, and 15; - 21-day cycle; 3-hour infusion on days 1, 8, and 15	Literature review, a detailed assessment of breast cancer forum discussions, and consultation with clinical experts
**4**	Beusterien et al. (15)	2	7 (2 - 4 levels)	Attributes and Levels: (1) 5-years iDFS: 76%, 83%, 88%, 95%; (2) Nausea: - Percent risk of nausea: 12%, 29%, 64%;- Percent risk of grade 3/4 nausea: 0%, < 1%, 5%; (3) Diarrhea: - Percent risk of diarrhea: 11%, 35%, 81%; - Percent risk of grade 3/4 diarrhea: 0%, 1%, 9%; (4) ECG monitoring: - Does not require routine ECG testing to assess heart function because there is no known risk of arrhythmia; - Requires ECG testing to assess heart function 3 times within the first 3 months of treatment to monitor the 6% risk of arrhythmia; (5) Neutropenia: - Percent risk of neutropenia: 1%, 24%, 66%;- Percent risk of febrile neutropenia: 0%, < 1%, 2%;6) Alopecia: 10%, 34%;(7) Dosing schedule: (A) One tablet, PO., QD for 5 years; (B) Two medicines initiated at the same time: -One tablet, PO, QD for 5 years; - One pill, PO, QD for 21 consecutive days followed by 7 days off; 28-day cycle is repeated for 2 years	Literature review, a detailed assessment of breast cancer forum discussions, and consultation with clinical experts

Abbreviations: iDFS, invasive disease-free survival; PO, orally; QD, daily; DCE, discrete choice experiment; ECG, electrocardiogram; GI, gastrointestinal.

In Study No. 1, respondents were presented with two labeled choice options, chemotherapy A and B, each varying in three identical attributes. Additionally, a "no chemotherapy" option was included, and respondents indicated their preferences and the strength of those preferences using a 7-point scale. Twelve attributes were reviewed: Alopecia, motor neuropathy, sensory neuropathy, myalgia/arthralgia, nausea and vomiting, fatigue, neutropenia, mucositis, hand-foot syndrome, diarrhea, efficacy, and treatment regimen.

In Study No. 2, fifteen choice sets were presented to the respondents, each containing two labeled hormone therapy options. Respondents indicated their preferences using a 9-point scale. This study utilized eight attributes, including prevention of breast cancer recurrence, osteoporosis, risk of endometrial cancer, joint and muscle pain, fluid retention, libido decrease, hot flashes, and regimen duration, each with two levels.

Study No. 3 employed five side effects containing two severity grades I/II and III/IV (peripheral neuropathy, motor neuropathy, myalgia, nausea, and fatigue), as well as alopecia and neutropenia. All side effect attributes had three levels, and administration regimen had seven levels.

Study No. 4 utilized five side effect attributes, including nausea, diarrhea, ECG monitoring, and neutropenia, as well as 5-years DFS and dosing schedule. Seven attributes were presented to the respondents in two choice sets.

Regarding the experimental design, all studies utilized the fractional factorial method to develop the choice profiles. Study No. 1, Study No. 2, and Study No. 3 employed labeled scenarios, while orthogonal arrays were used in all studies to conduct choice sets. Concerning statistical analysis, Study No. 1 and Study No. 3 employed Ordinary Least Square (OLS), Study No. 2 used logistic regression, and Study No. 4 utilized HB (Hierarchical Bayesian) Logistic Regression as an analytical tool.

### 3.3. Risk of Bias Assessment

According to the risk of bias checklist outlined in section 2.5, the predominant limitation observed across all studies was the insufficient reporting of methodological aspects. None of the four studies provided any information about the construction of tasks. Additionally, Study No. 2 and Study No. 4 did not provide any information about developing the experimental design (Table 3). 

**Table 3.** Risk of Bias Assessment

Study	Research Question	Attributes and Levels	Construction of Tasks	Experimental Design	Preference Elicitation	Instrument Design	Data Collection	Statistical Analyses	Results and Conclusions	Study Presentation
**Beusterien et al. 2012 (** 12 **)**	Yes	Yes	No	Yes	Yes	Yes	Yes	Yes	Yes	Yes
**Wouters et al. 2013 (** 13 **)**	Yes	Yes	No	No	Yes	Yes	Yes	Yes	Yes	Yes
**Beusterien et al. 2014 (** 14 **)**	Yes	Yes	No	Yes	Yes	Yes	Yes	Yes	Yes	Yes
**Beusterien et al. 2021 (** 15 **)**	Yes	Yes	No	No	Yes	No	Yes	Yes	Yes	Yes

### 3.4. Study Participants and Diagnosis

Study No. 1 and Study No. 4 were conducted in the USA. Study No. 1 included patients above 18 in any stage with previous chemotherapy during the last five years, while Study No. 4 comprised patients in stages 2-3 with a history of chemotherapy over the last five years. Study No. 2 was conducted in the Netherlands with a patient group receiving hormone therapy, while Study No. 3 was completed in Canada with a patient group receiving chemotherapy in all stages (Table 1). 

### 3.5. Development of Attributes and Levels

All studies employed systematic review as one of the attribute selection methods. Study No. 2 also utilized a focus group as another tool to select attributes and levels from the patient's point of view. Additionally, consultations with clinical experts were conducted in Study No. 1, Study No. 3, and Study No. 4 to assist with attribute and level selection.

### 3.6. Survey Design

Table 2 displays the number of attributes in each study, ranging from a minimum of seven attributes to a maximum of 12. Furthermore, the minimum number of levels for attributes is two, while the maximum reaches seven. This variation is observed given that the average number of attributes is five, and the average number of levels for each attribute is 3.5. All four studies elicited preferences using fractional factorial methods. Study No. 2 employed an orthogonal design for developing experimental design, although other studies did not clarify this. All studies utilized Sawtooth software for experimental design, with only Study No. 4 employing SAS v.9.3 and SPSS v.25.0 software packages in addition to Sawtooth.

Regarding the number of choice tasks presented to the respondents, only Study No. 2 mentioned that each respondent answered the 15 choice tasks. None of the studies mentioned blocking.

Table 4 illustrates the sample size for each study, with a minimum of 102 and a maximum of 300 participants. Data collection methods varied: Study No. 1, Study No. 3, and Study No. 4 utilized online data collection, while Study No. 2 employed both online and face-to-face interviews.

**Table 4.** Experimental Design

Study	Study Type	Sample Size	Population	Tumor Staging	Age	Blocks	N Tasks/Patient	Estimation Method	Pilot Study
**Beusterien et al. (** 12 **)**	Online	108	Women with the USA residency	1 - 4	50.43 ± 8.56	N/A	N/A	OLS	No
**Wouters et al. (** 13 **)**	Face-to-face, online	241	Patients in pharmacy, hospital	N/A	57.2 ± 10	N/A	15	Linear regression	No
**Beusterien et al. (** 14 **)**	Web survey	102	Female breast cancer	Any stages	54 ± 11.3	N/A	N/A	OLS	No
**Beusterien et al. (** 15 **)**	Online	300	HR+, HER2- breast cancer	2 or 3	58.9 ± 10.1	N/A	N/A	HB logistic regression	Yes

Abbreviations: N/A, not applicable; OLS, ordinary least square; HB, hierarchical bayesian.

All attributes were extracted by one of the research team members (AH) and categorized into three groups: Efficacy, side effects, and treatment regimen. Study No. 2 included only side effects and treatment regimen attributes, whereas the other three studies encompassed all three groups.

### 3.7. Analysis Method

Table 4 outlines the analysis methods for each study. Study No. 1 and Study No. 3 employed the OLS method for preference elicitation, Study No. 2 used logistic regression, and Study No. 4 utilized HB logistic regression as the analysis method.

### 3.8. Extracted Attributes

Treatment regimen, side effects, and 5 years DFS are the extracted attributes that will be used in the next step to conduct the DCE study.

## 4. Discussion

Four DCE studies in breast cancer have been reviewed, with all four eliciting patient preferences except Study No. 4, which also elicited physician and policymakers' preferences. Using systematic methods to choose attributes is crucial for conducting preference studies, especially considering the varied levels of knowledge and signs among the patient population. Thus, systematic studies like qualitative research (such as focus groups and expert panels) and SR (systematic review) are necessary. All reviewed studies employed SR as one of the attribute selection methods. Study No. 2 also utilized a focus group method, while other studies used consultations with experts as another qualitative method for defining attributes.

Different attributes were reviewed in each study based on the study concept. Efficacy, treatment regimen, and adverse effects were among the most reviewed attributes. Disease-free survival, an efficacy indicator, was categorized into various percentage levels. Treatment regimens were developed based on available guidelines, with different levels. Since chemotherapy entails various side effects, a wide range of side effects were included, such as sensory and motor neuropathy, alopecia, nausea and vomiting, fatigue, fever and neutropenia, mucositis, arthralgia, hyperlipidemia, osteoporosis, fatty liver, and endometrial cancer.

Most of the studies utilized Sawtooth software for experimental design but did not provide further information about the details of the experimental design. In preference studies, it is crucial to use an appropriate experimental design to obtain valid results, so it is important to mention the details of the design. Only one of the studies explains the data analysis and software packages. Therefore, presenting information about the details of the software and the method of developing the experimental design are essential parts of preference research.

Study No. 2 incorporated the "indifferent" option in the questionnaire design, while Study No. 3 used the "no preference" option. However, in realistic situations, patients are often required to choose a treatment option, so these options may not accurately reflect real-life decision-making. Respondents may choose these options to avoid difficult decisions. Therefore, in future research, it is important to explore methods that reflect reality more accurately to estimate preferences more realistically.

A wide range of sample sizes has been used in different studies, but none of them mention the method of estimating the sample size. Since the sample size can influence the final results and the study's validity, it is essential to follow a predetermined method for choosing a sample size, which can be found in previous research.

Some attributes, such as outcomes and side effects, are probabilistic parameters, and the target patient population may not fully understand these parameters, leading to unreliable responses. To make the questionnaire more understandable for such probabilistic parameters, some researchers use pictures or graphic design and convert them into deterministic parameters to simplify decision-making for respondents. None of the reviewed studies addressed this point, and only one reported the respondents' response rate.

### 4.1. Conclusions

Since patients are at the core of treatment, involving them in the treatment procedure and considering their preferences in choosing treatment, especially in chronic diseases, has become a hot topic in health outcome research. DCE research is one of the most common preference elicitation methods used today. The results of DCE studies would help in choosing treatments, calculating willingness to pay (WTP), supporting policymakers in finding the best policies, and aiding pharmaceutical companies in their research and development efforts to develop new products or enhance existing ones. Designing a graphical questionnaire can make it more understandable and easy to answer. Additionally, it is important to mention how the standard method selects the sample size. As a result, this research finds that efficacy, side effects, and treatment regimen are attributes used in preference research in hormone receptor-positive, HER2-negative early-stage breast cancer.

### 4.2. Limitations

We cannot quantify the results due to the wide range of extracted information. Additionally, most studies focused more on side effects and provided less information about cost and efficacy, resulting in a predominance of data on side effects. Furthermore, some research was supported by pharmaceutical companies, raising the possibility of influencing the results. This study serves as a guide for future research.

## Data Availability

The dataset presented in the study is available on request from the corresponding author during submission or after publication.
